# Evaluation of Candidate Reference Genes for Gene Expression Normalization in *Brassica juncea* Using Real Time Quantitative RT-PCR

**DOI:** 10.1371/journal.pone.0036918

**Published:** 2012-05-11

**Authors:** Ruby Chandna, Rehna Augustine, Naveen C. Bisht

**Affiliations:** National Institute of Plant Genome Research, Aruna Asaf Ali Marg, New Delhi, India; Kyushu Institute of Technology, Japan

## Abstract

The real time quantitative reverse transcription PCR (qRT-PCR) is becoming increasingly important to gain insight into function of genes. Given the increased sensitivity, ease and reproducibility of qRT-PCR, the requirement of suitable reference genes for normalization has become important and stringent. It is now known that the expression of internal control genes in living organism vary considerably during developmental stages and under different experimental conditions. For economically important Brassica crops, only a couple of reference genes are reported till date. In this study, expression stability of 12 candidate reference genes including *ACT2*, *ELFA*, *GAPDH*, *TUA*, *UBQ9* (traditional housekeeping genes), *ACP*, *CAC*, *SNF*, *TIPS-41*, *TMD*, *TSB* and *ZNF* (new candidate reference genes), in a diverse set of 49 tissue samples representing different developmental stages, stress and hormone treated conditions and cultivars of *Brassica juncea* has been validated. For the normalization of vegetative stages the *ELFA, ACT2*, *CAC* and *TIPS-41* combination would be appropriate whereas *TIPS-41* along with *CAC* would be suitable for normalization of reproductive stages. A combination of *GAPDH*, *TUA, TIPS-41* and *CAC* were identified as the most suitable reference genes for total developmental stages. In various stress and hormone treated samples, *UBQ9* and *TIPS-41* had the most stable expression. Across five cultivars of *B. juncea,* the expression of *CAC* and *TIPS-41* did not vary significantly and were identified as the most stably expressed reference genes. This study provides comprehensive information that the new reference genes selected herein performed better than the traditional housekeeping genes. The selection of most suitable reference genes depends on the experimental conditions, and is tissue and cultivar-specific. Further, to attain accuracy in the results more than one reference genes are necessary for normalization.

## Introduction

Gene expression analysis is extremely important in many fields of biological research. Understanding the expression pattern of genes provides a useful mean of studying the complex regulatory networks occurring in living organism. Among the widely used methods to measure the levels of gene expression, real time quantitative reverse transcription PCR (qRT-PCR) represents a suitable technology [1]. Being an efficient, sensitive, and reliable method, qRT-PCR provides a rapid mean towards simultaneous measurement of gene expression across different samples [2]. Since this platform is relatively simple coupled with a high level of sensitivity, qRT-PCR is rapidly being adopted as a standard method for performing in-depth expression analysis of number of target genes. For accurate and reliable analysis of target gene expression, normalization of qRT-PCR data with suitable internal reference gene(s) is required [3]. Normalization is essential to correct the non-specific variations arising because of the difference in amount of template used and its quality that can affect the efficiency of the qRT-PCR reactions [4]. Normalization also allows the direct comparison of normalised transcript expression levels between samples [5]. An ideal reference gene should express at constant level in all tissues and at all developmental stages, regardless of the experimental conditions or treatments [6], [7].

Commonly used reference genes are mostly cellular maintenance genes (also known as housekeeping genes), which are involved in basic and ubiquitous cellular processes such as components of the cytoskeleton, glycolytic pathway, protein folding, protein degradation, synthesis of ribosome subunits. Most frequently used housekeeping genes including β-actin (*ACT*), α-tubulin (*TUA*), ubiquitin (*UBQ*), glyceraldehde-3-phosphate dehydrogense (*GAPDH*), *18S* or *26S* ribosomal RNA and elongation factors (*EF*) have been validated as suitable internal control genes in many plants [5], [8]–[11]. These genes were assumed to be expressed constitutively and also have constant expression levels between different samples. However, there are evidences that transcripts levels of housekeeping genes vary considerably across the developmental stages and under variable conditions [6]. This variability in expression across experimental samples may be because these housekeeping genes not only participate in basic cell metabolism but also in other cellular processes [12]. Therefore, selecting multiple stably expressed reference genes, other than the commonly used housekeeping genes need to be considered for accurate normalization of gene expression studies [13].

Recognising the importance of validation of reference gene(s) for normalization of qRT-PCR data initiated the development of number of software packages such as geNorm [6], [13] and NormFinder [4] Usage of these statistical algorithms have greatly simplified the selection of appropriate reference genes by calculating the expression stability and determining the optimal number of candidate reference genes required for normalization under specific conditions in various organisms, including plants [14], [15]. A number of attempts for reference gene validation have been reported in plants such as rice [16], chickpea [10], potato [8], soybean [9], [17], tomato [18], chrysanthemum [11], grape [19], cabbage [20], wheat [21], *Brassica napus* [22] and poplar [23].

The *Brassica* species have diverse characteristics and are of great agronomic importance as vegetables, condiments, fodder, and oil crops. *Brassica* crops are globally the third most important sources of vegetable oil after soybean and groundnut [24]. *Brassica juncea* (brown or Indian mustard) is an important oilseed crop cultivated mainly in Indian sub-continent besides some parts of east Europe, Africa, Canada and China. Only a limited number of gene expression studies have been carried out in *B. juncea*, wherein *ACT* and *18S* are the commonly used reference genes [25]–[29].

Comparison of several candidate reference genes in Brassica crops, particularly in *B. juncea*, is not yet reported, thereby limiting our knowledge about the choice of best reference gene which could be used for normalization of gene expression across developmental stages and variable growth conditions. In present study, we have compared the performance of 12 candidate reference genes (consisting of five commonly used housekeeping genes of plants, and seven new candidate reference genes selected from *B. rapa* and *B. napus* microarray platforms) in 49 diverse samples of *B. juncea*, broadly categorized into five distinct experimental sets. Our results reveal that new reference genes are more stably expressed than the traditionally used housekeeping genes across all the five experimental sets. Further, combination of most stable reference genes provides a more accurate and reliable mean of normalization during qRT-PCR analysis.

## Results

### Selection of Candidate Reference Genes and Primer Design

A total of 12 candidate reference genes, including five traditional housekeeping genes namely, actin2 (*ACT2*), elongation factor 1B (*ELFA*), glyceraldehyde-3-phosphate- dehydrogenase (*GADPH*), α-tubulin (*TUA*) and ubiquitin 9 (*UBQ9*) and seven new reference genes, acyl carrier proteins (*ACP*), clathrin adaptor complex (*CAC*), sucrose non fermenting-1 protein kinase (*SNF*), tonoplastic intrinsic proteins-41 (*TIPS*-*41*), trans membrane proteins (*TMD*), tryptophan synthase-β (*TSB*) and zinc finger protein (*ZNF*) were used in this study. The new candidate reference genes were selected on the basis of their stable expression profiles across developmental stages and during abiotic stress conditions, as determined using microarray data of *B. rapa* (www.brassica.info/resource/trancriptomics.php) [30] and *B. napus* (www.rapeseed.plantsignal.cn) [31], [32] and analysed using *A. thaliana* gene expression tool (www.jsp.weigelworld.org) [33] (File S1, S2 and S3).

Since, sequence information of *B. juncea* is very limited, we used publicly available gene sequences from Arabidopsis and related Brassica species to design the gene specific primers. The Brassica specific expressed sequence tags (EST) and genome survey sequences (GSS) of these candidate reference genes were largely obtained from the Brassica genome gateway (http://brassica.bbsrc.ac.uk) [34] and the recently available *B. rapa* genome portal (http://brassicadb.org/brad) [35], by providing Arabidopsis complementary DNA sequence (CDS) as a query. To ensure gene amplification in *B. juncea*, the Arabidopsis and Brassica sequences of each candidate gene were aligned together and the primers were designed from the consensus regions of the aligned sequences, preferably spanning an intron (File S4).

### Verification of Primer Specificity and PCR Efficiency Analysis

In order to determine specificity of primers designed in the current study, agarose gel electrophoresis and melting curve analyses were performed following the qRT-PCR experiment on seedling stage of *B. juncea* L. cv. Varuna. All the primer pairs amplified single PCR product of expected size (File S5) and the specificity of amplicon was confirmed by the presence of single peak during melt curve and sequencing analysis (File S6 and S7). A standard curve was generated using 10-fold serial dilutions of cDNA to calculate the gene specific PCR efficiency. The slopes of the standard curves were used to calculate the correlation coefficient (R^2^) and PCR efficiency (File S8). The linear R^2^ for all the primers ranged between 0.994–0.999 over 1000 fold of cDNA dilution. Further, PCR efficiencies of primers ranged from 94%–106% (Table 1).

**Table 1:** Surveyed references genes with their amplification and expression characteristics in *B. juncea.*

Internal Reference	Primers (F/R)	Amplicon length (bp)	T_m_ (°C)	*PCR efficiency (%)*	*Regression coefficient (R^2^)*	*Covariance* *(%)*
***ACP***	5′- GTTGATTATGGGAAGAAGTCTAAGCT	110	82.98	99.6	0.998	8.34
	5′- TTGTAAGGCTCTACAACAGCAGTA					
***ACT2***	5′-TGGGTTTGCTGGTGACGAT	290	83.72	104.3	0.994	8.07
	5′- TGCCTAGGACGACCAACAATACT					
***CAC***	5′- CAATCGATTGCTTGGTTTGG	110	78.33	94.5	0.990	4.34
	5′- CAAGTCCAAGATTTCTTCTCTCC					
***ELFA***	5′- CCAAGAATGGGCTTTATGC	130	81.18	99.0	0.998	14.57
	5′- GTGATAGAGTGTCCAACAAGGTAAGTA					
***GAPDH***	5′- TCAGTTGTTGACCT CACGGTT	100	81.48	102.3	0.999	17.77
	5′- CTGTCACCAACGAAGTCAGT					
***SNF***	5′- CAAAGTCAACTGTTGGTACTCCTG	150	79.98	103.1	0.985	4.98
	5′- ATGGATATGCATCAACCAACAT					
***TIPS-41***	5′- TGAAGAGCAGATTGATTTGGCT	100	76.99	103.4	0.999	4.96
	5′- ACACTCCATTGTCAGCCAGTT					
***TMD***	5′- ACTCAATTCTATCTCCGCCTCT	85	81.03	97.4	0.999	7.53
	5′- AACAGAGCTCCGCATATTCC					
***TSB***	5′- AAGTACGTCCCCGAAACTCTAATG	145	82.23	106.3	0.995	7.23
	5′- CTGCGAAGTAGAGAGGACTTTC					
***TUA***	5′- GCTGGGTCACTCCAGATTTTG	80	77.58	99.7	0.998	6.26
	5′- CCATCGCCTTGTCTGCAAG					
***UBQ9***	5′- GAAGACATGTTCCATTGGCA	160	80.73	99.5	0.997	12.27
	5′- ACACCTTAGTCCTAAAAGCCACCT					
***ZNF***	5′- ATTTTCAGGCGGTTTATGGC	150	82.07	99.8	0.974	11.14
	5′- CTCTTGCTTTCTTCTTGGCGT					
# ***BjDREB2***	5′-TGTATGAAAGGCAAAGGAGGA	130	85.0	96.56	0.985	−
	5′-GAAAGTACCAAGCCAAAGCCT					

# - used for normalization validation during abiotic stress conditions.

### Expression Profiling of *B. juncea* Reference Genes

**Figure 1:**
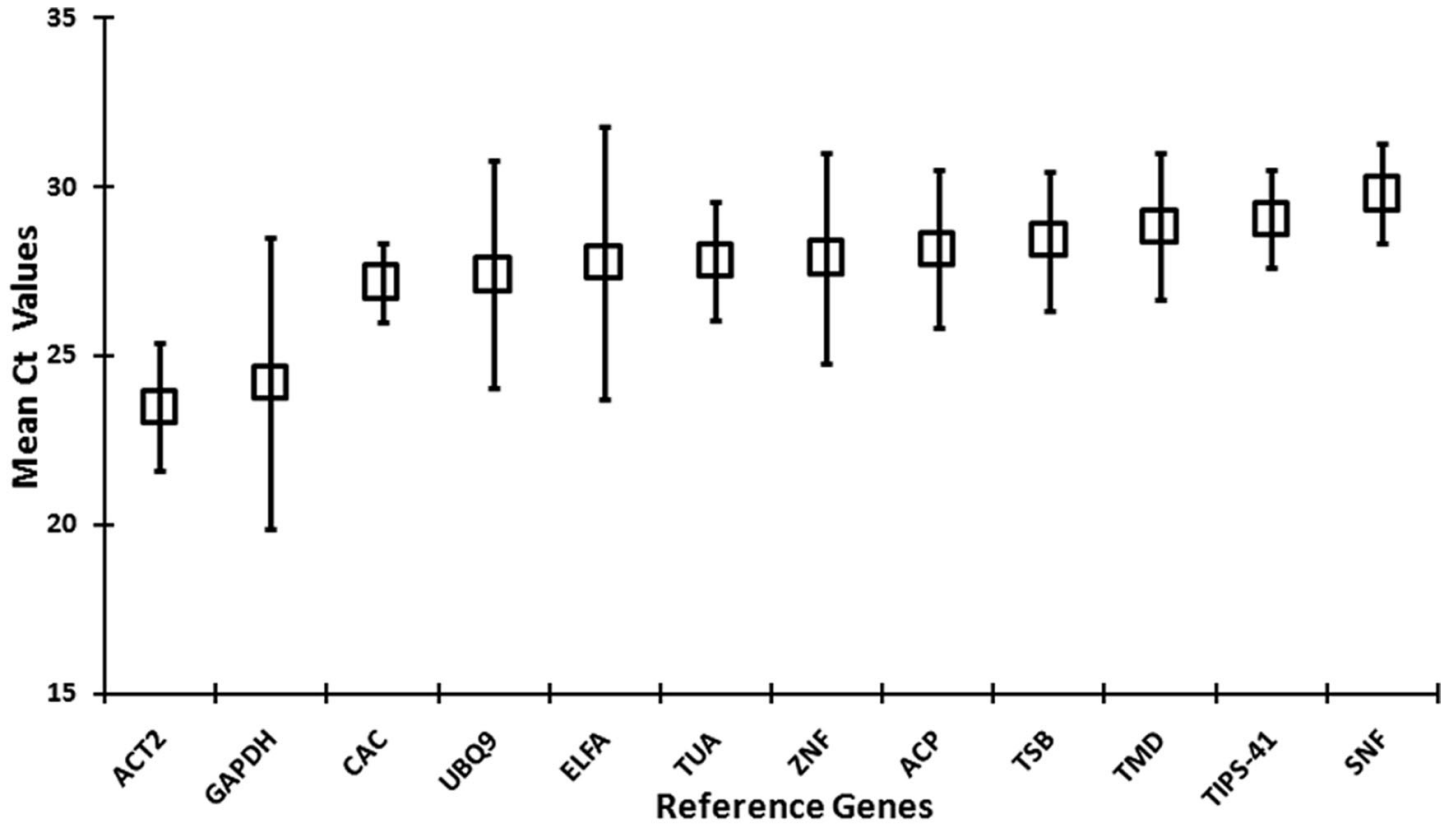
Expression levels of 12 candidate reference genes across all the five experimental sets of *B. juncea*. The boxes represent mean Ct values and bars correspond to the standard deviation. The Ct values were calculated on 1∶50 diluted cDNA samples.

A real-time qRT-PCR assay, based on SYBR Green detection, was designed for transcript profiling of the 12 candidate reference genes (*ACP*, *ACT2*, *CAC, ELFA*, *GAPDH*, *SNF*, *TIPS-41*, *TMD*, *TSB*, *TUA*, *UBQ9* and *ZNF*) in 49 diverse samples of *B. juncea* (Table 1). In order to minimize the variability associated with qRT-PCR analysis, all RNA samples were adjusted to same concentration and quality pass prior to their conversion into cDNA (File S9). The expression level of the candidate genes obtained during qRT-PCR experiments are presented as threshold cycle (Ct) values. The 12 reference genes used in the current study showed relatively wide ranges of Ct values across 49 samples of *B. juncea*. The mean Ct values of reference genes ranged from 22.02–29.36 (Figure 1). *GAPDH* was the most abundant reference gene of the set (mean Ct  = 22.02), whereas *TMD* was the least abundant reference gene (mean Ct  = 29.36). Interestingly, three new candidate reference genes *CAC*, *TIPS-41* and *SNF* showed least expression variation (Co-variance, CV of 4.34, 4.96 and 4.98, respectively); while the commonly used housekeeping genes *GAPDH* and *ELFA* (CV of 17.77 and 14.57, respectively) had the most variable expression profile across all the 49 experimental samples (Figure 1).

In order to perform in-depth expression analysis of candidate reference genes, the 49 diverse samples were categorized under five experimental sets comprising of defined development or condition-specific samples of *B. juncea* (Table 2). The first experimental set comprised of six vegetative stages (cotyledon, seedling, young leaf, mature leaf, stem and roots), whereas the second set included six reproductive stages (flower bud, flower, pod 5 days post anthesis (dpa), pod 10 dpa, pod 15 dpa and pod 30 dpa). In the third experimental set, all the 12 developmental stages of *B. juncea* (both vegetative and reproductive stages as mentioned above) were analysed together. The fourth set consisted of 12 samples treated with different abiotic stress conditions and hormones. The fifth experimental set included the vegetative stages of Indian and east-European *B. juncea* cultivars (Pusa Bold, Kranti, Donskaja, Early Heera-2, and Zem 84500).

**Table 2:** Expression levels of 12 reference control genes across five experimental sets of *B. juncea* using geNorm.

Genes	VegetativeStagesMean Ct ± SD*	Reproductive StagesMean Ct ± SD	Total Development stagesMean Ct ± SD	TreatmentsMean Ct ± SD	CultivarsMean Ct ± SD
***ACP***	27.6±1.4	25.8±0.8	26.7±1.5	26.7±1.1	29.5±2.3
***ACT2***	22.4±0.6	23.9±2.7	23.1±2.1	23.4±0.6	23.5±2.2
***CAC***	27.3±0.9	26.0±0.6	26.7±0.9	26.9±0.7	27.4±1.4
***ELFA***	29.3±0.6	29.6±2.3	29.4±1.6	29.7±1.1	25.9±4.8
***GAPDH***	21.4±1.6	19.8±0.6	20.6±1.4	21.0±1.3	27.3±3.9
***SNF***	28.5±0.9	28.9±1.4	28.7±1.1	27.7±0.7	29.4±1.5
***TIPS-41***	28.7±0.9	27.6±0.8	28.2±0.9	28.2±0.6	29.6±1.6
***TMD***	29.3±0.5	30.1±1.8	29.7±1.3	29.1±0.7	28.6±2.8
***TSB***	28.0±1.3	27.2±2.8	27.6±2.1	28.7±0.8	28.6±2.4
***TUA***	28.1±1.6	28.4±0.7	28.3±1.2	27.6±0.9	27.9±2.1
***UBQ9***	26.7±3.9	28.9±1.9	27.8±3.1	25.6±0.5	29.3±2.1
***ZNF***	24.9±3.3	22.4±1.1	23.7±2.7	27.9±0.6	29.8±1.8

* - mean of Ct values from all analyzed samples in individual experimental sets along with the standard deviations (SD) observed.

It was observed that the tissue type and experimental conditions does affect the expression of reference genes. In vegetative stages, *TMD*, *ELFA* and *ACT2* showed the least expression variation whereas a large variation in the expression levels of *UBQ9* and *ZNF* was observed (Table 2). In reproductive stages *TSB, ACT2* and *ELFA* showed large variation in their expression profiles, whereas *CAC* and *GAPDH* were the least variable transcripts. Interestingly, across total developmental stages, the two new reference genes *TIPS-41* and *CAC* emerged as most stable transcripts showing least variation and *UBQ9* and *ZNF* were the most variable transcripts (Table 2). In contrast, the expression of *UBQ9* was least variable in stress and hormone treated samples, whereas expression of *GAPDH* varied widely in various treated samples (Table 2). A significant variation in the expression of *ELFA* and *GAPDH* was also observed across the cultivars, while *CAC* and *TIPS-41* showed least expression variation (Table 2). In general, expression of all the 12 reference genes selected herein showed a large variation across the five cultivars of *B. juncea.* This probably reflects the presence of allelic variation in these genes across the *B. juncea* cultivars. Thus, transcript levels of none of the candidate reference genes remain constant throughout the developmental stages, stress and hormone treatments and across the *B. juncea* cultivars.

### Gene Expression Stability Analysis and Ranking of *B. juncea* Reference Genes

**Figure 2:**
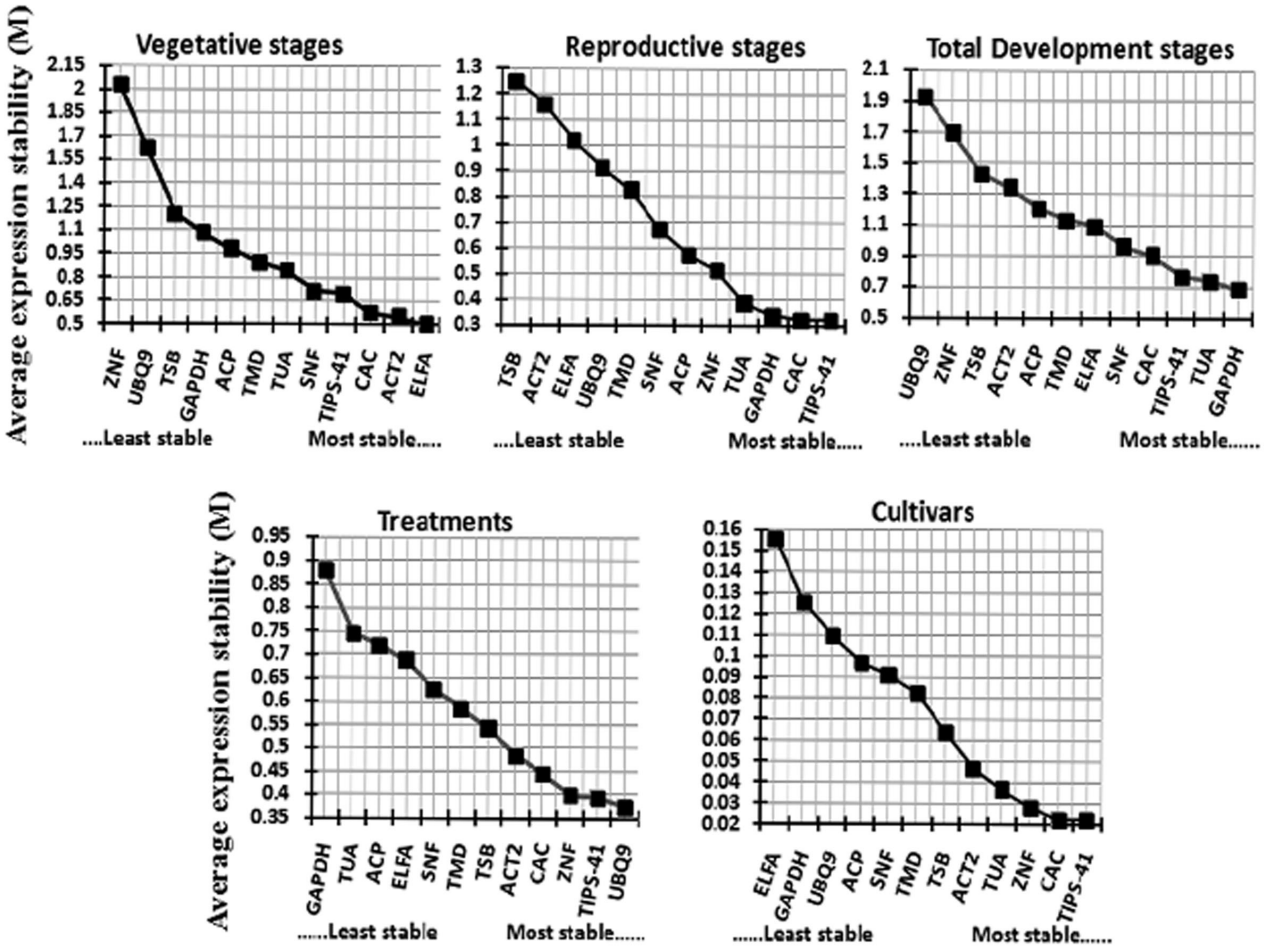
Gene expression stability of the 12 candidate genes of *B. juncea* as predicted by geNorm. Mean expression stability (M) following stepwise exclusion of the least stable gene across all the samples within an experimental set. The least stable genes are on the left, and the most stable on the right.

The expression stability of 12 candidate reference genes, across five experimental sets, was measured and ranked using two different programs namely geNorm [6] and NormFinder [4]. Based on geNorm analysis, *ELFA*, *ACT2*, *CAC*, and *TIPS-41* (in order) were identified as the most stable genes across the vegetative stages whereas *TIPS-41*, *CAC*, *GAPDH* and *TUA* remained the most stable genes across the reproductive stages (Figure 2). When all the 12 samples were analysed together, *GAPDH*, *TUA*, *TIPS-41* and *CAC* were the most stable genes, while *UBQ9* and *ZNF* were least stable transcripts, in order. It is interesting to note that most stable housekeeping genes of vegetative stage (*ELFA* and *ACT2*), were not among the most stable reference genes of the total developmental set in *B. juncea*. Further, in response to various stress and hormone treatments, *UBQ9*, *TIPS-41*, *ZNF* and *CAC* were the most stable genes whereas across the *B. juncea* cultivars, *TIPS-41*, *CAC*, *ZNF* and *TUA* were identified as the most stable genes, in order. The commonly used housekeeping gene *GAPDH* was, however, found to be among the least stable transcript in treated samples as well as across the cultivars of *B. juncea* (Figure 2).

The NormFinder also ranked *ELFA, TIPS-41, CAC* and *ACT2* as the most stable reference genes in vegetative stages (in order), whereas in reproductive stages *TIPS-41*, *CAC*, *ZNF* and *TMD* were identified as the most stable reference genes (Table 3). Across total developmental stages *GAPDH*, *TIPS-41*, *ELFA* and *TUA* were the most stable genes while *ZNF* and *UBQ9* were the least stable genes (Table 3). Both, *CAC* and *TIPS-41* also emerged as most stable reference genes in stress and hormone treated samples and also across the *B. juncea* cultivars. Thus using both programs, similar genes were identified as stable reference genes across all the five experimental sets although the gene expression stability rankings for some reference genes were slightly altered.

**Table 3:** Expression stability of the 12 reference control genes of *B. juncea* as calculated by Normfinder.

Rank	Vegetative stages		Reproductive stages		Total Development stages		Treatments		Cultivars	
	Gene	Stability	Gene	Stability	Gene	Stability	Gene	Stability	Gene	Stability
1	*ELFA*	0.223	*TIPS-41*	0.443	*GAPDH*	0.526	*TIPS-41*	0.304	*CAC*	0.179
2	*TIPS-41*	0.286	*CAC*	0.542	*TIPS-41*	0.540	*CAC*	0.361	*TIPS-41*	0.253
3	*CAC*	0.335	*ZNF*	0.581	*ELFA*	0.688	*UBQ9*	0.407	*ZNF*	0.278
4	*ACT2*	0.505	*TMD*	0.608	*TUA*	0.820	*SNF*	0.444	*TUA*	0.341
5	*ACP*	0.784	*UBQ9*	0.671	*CAC*	0.954	*ZNF*	0.461	*ACT2*	0.410
6	*SNF*	0.786	*TUA*	0.773	*TMD*	0.989	*TSB*	0.513	*TSB*	1.241
7	*TMD*	0.835	*SNF*	0.971	*SNF*	1.025	*ACT2*	0.556	*TMD*	1.248
8	*GAPDH*	1.045	*GAPDH*	1.011	*ACP*	1.050	*ELFA*	0.658	*UBQ9*	1.554
9	*TUA*	1.120	*ELFA*	1.031	*TSB*	1.191	*TMD*	0.661	*SNF*	2.120
10	*TSB*	1.534	*ACP*	1.265	*ACT2*	1.239	*ACP*	0.690	*ACP*	3.134
11	*UBQ9*	3.572	*ACT2*	1.553	*UBQ9*	1.820	*TUA*	0.729	*GAPDH*	3.961
12	*ZNF*	4.133	*TSB*	1.568	*ZNF*	2.055	*GAPDH*	1.492	*ELFA*	4.724

### Optimal Number of Reference Gene for Normalization Across the Experimental Sets

**Figure 3:**
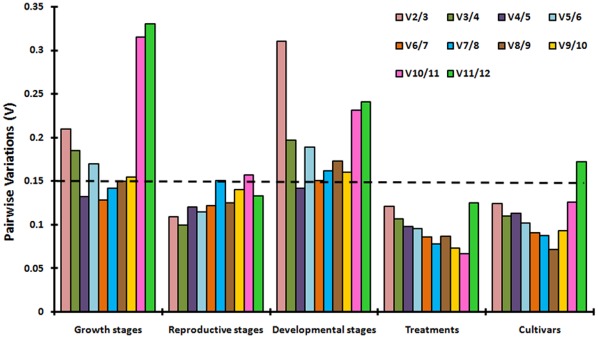
The optimal number of reference genes required for effective normalization in each experimental sets of *B. juncea*. The pairwise variation (Vn/Vn+1) was analyzed between normalization factors NFn and NFn+1 by geNorm program to determined the optimal number of reference genes.

The geNorm software was further used to calculate the optimal number of reference genes necessary for normalization across different sets of experiment. The pairwise variation (Vn/Vn+1) between sequential normalization factors, NFn and NFn+1 was used to determine the number of genes required for reliable normalization [6]. As shown in Figure 3, differences in the expression stability values of the candidate reference genes were less marked in reproductive stages, than in other series (Figure 3). The V2/3 value for reproductive stage was 0.108 (geNorm V <0.15 when comparing a normalization factor based on the two or three most stable targets), so *CAC* together with *TIPS-41* would be sufficient for normalization purpose in reproductive stages (Figure 3). In vegetative stages, V4/5 value was 0.132 thereby suggesting that the optimal number of reference targets would be four, namely *ELFA, ACT2, CAC* and *TIPS-41*. However on analysing all the developmental stages together, four reference genes namely *GAPDH*, *TUA*, *TIPS-41* and *CAC* should be considered (V4/5 value  = 0.137). In treated samples, the pair of *TIPS-41* and *UBQ9* produced a V2/3 value of 0.12, therefore these two candidate reference genes can be used for normalization for a wide range of stressed and hormone treated tissue samples in *B. juncea*. Across cultivars, *TIPS-41* and *CAC* could be the choice of optimal reference genes (V2/3 value of 0.124). Thus across all the five experimental sets of *B. juncea*, the two new candidate reference genes namely *TIPS-41* and *CAC* were identified as the most suitable reference genes for normalization in gene expression studies.

### Reference Gene Validation

**Figure 4:**
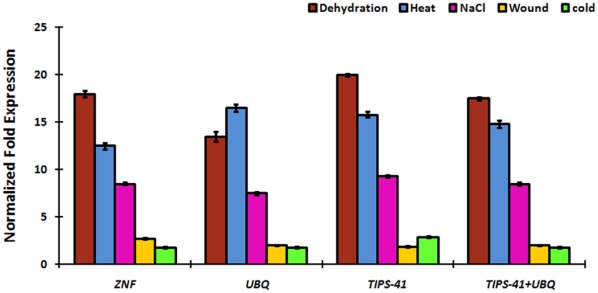
Relative quantification of *BjDREB*-2 expression using identified stable reference genes and their combination for normalization under various stress conditions.

To validate the selection of candidate reference genes for normalization, we analyzed the normalized fold expression of *BjDREB2* (a dehydration responsive element binding proteins-2 homolog of *B. juncea*). DREB2 is a transcription factor that imparts stress endurance to plants and plays a crucial role in providing tolerance to heat, dehydration, wounding and salt stresses. *UBQ9*, *TIPS-41* and *ZNF* reference genes identified as the most stable genes using geNorm analysis among all stress treated samples, were further tested for normalization. The transcript abundance of *BjDREB2* increased in dehydration stressed sample when normalized using all the three genes independently, although at different level (Figure 4). The expression pattern of *BjDREB2* transcript was higher in heat stress in case of *UBQ9*, whereas the increased expression pattern was observed in dehydration samples using either *ZNF* or *TIPS-41* as the reference gene. However, when the expression of *BjDREB2* was normalized using a combination of *UBQ9* and *TIPS-41*, identified by geNorm as most stable reference genes, the fold expression of *BjDREB2* was highest in dehydration stressed sample. This data clearly suggests that the use of more than one reference genes for normalization provides more accurate representation of target gene expression tested across the variable experimental conditions. As expected, the *BjDREB2* expression remains unaltered in hormone treated samples (data not shown).

## Discussion

Quantitative real time PCR has become a powerful technique for gene expression studies, because of its high throughput, sensitivity and accuracy [2]. The choice of stably expressed reference genes for normalization is the paramount to accurate interpretation of the results. The normalization takes care of the variation introduced by the quantity and quality of input RNA, its cDNA conversion and the various steps involved in the qRT-PCR assay. Normalization studies with multiple reference genes validated for their expression stability is required for reliable gene expression results, as no single reference gene in plants has been shown to have a stable expression during variable experimental conditions including different samples/treatments. The usage of statistical algorithms, such as geNorm and NormFinder, has greatly simplified the selection and validation of reference genes by calculating the expression stability of reference genes as well as determining the number of reference genes required for accurate normalization across the experimental conditions tested [4], [6].

This study describes a comprehensive analysis on the validation of 12 candidate reference genes (including five commonly used housekeeping genes of plants and seven new candidate reference genes) in 49 diverse samples of *B. juncea*, divided broadly into five experimental sets. Our analysis based on geNorm and NormFinder algorithms indicated that the choice of reference genes for normalization should be experiment and stage-specific. For example, across total developmental stages of *B. juncea* (including both vegetative and reproductive stages) four genes namely *GAPDH*, *TUA*, *TIPS-41* and *CAC* are ideal for normalization factor. However, when the total developmental stages were subdivided into two distinct experimental sets viz., the vegetative and reproductive stages, different sets of candidate reference genes appeared to be the best for normalization in each stage (Figure 2; Table 3). For vegetative stages, the four reference genes viz., *ELFA*, *ACT2*, *CAC* and *TIPS-41* are ideal for calculating normalization factor, however for reproductive stages *TIPS-41* and *CAC* are enough for calculating the normalization factor. Since seed development is an economically important and distinct phase in Brassica crops, identification of suitable reference genes, particularly in reproductive stages will greatly assist in performing in-depth expression analysis of seed-specific genes in the said crop plants.

Some of the novel candidate reference genes selected in the current study performed better than the traditional housekeeping genes under each experimental set. *TIPS-41* (Tonoplast intrinsic proteins) and *CAC* (Clathrin adaptor complex), were identified amongst the list of most stable reference genes in all the five experimental sets of *B. juncea* tested in this study. *TIPS*-*41* (or *TIP41*) is one of the top ranked reference genes identified in the both vegetative and reproductive stages of *B. juncea* and *B. napus*, probably reflecting its similar and stable transcription regulation across the Brassica crops [22]. Previous studies in selection of reference genes during tomato development, *Fagopyrum esculentum* developmental stages and soybean under varied light regimes [9], [18], [36] also identified *TIPS-41* as the most stable reference gene for the vegetative sample. Similarly, *CAC* has also proved to be the best candidate for normalization in banana fruit, tomato, coffee, buckwheat and *Cucurbita pepo* studies [18], [37]–[39].

In current study, we tested the expression stability of commonly used housekeeping genes like *GAPDH*, *UBQ*, *ACT*, *ELFA* and *TUA* that have been previously described as “candidate controls” in various plant studies. Some of these housekeeping genes qualify among the best reference genes under specific experimental sets of *B. juncea*, however none of them was found to be the suitable reference gene across all the five experimental sets of *B. juncea*. For example, *UBQ9* was found to be the most appropriate gene in stress and hormone treated samples while expression stability of *UBQ9* ranked late in different sets of samples including total developmental stages (Figure 2; Table 3). Our findings are in accordance with the previous studies, wherein *UBQ9* and its putative homologues were the least stably expressed genes during total developmental stages in rice, soybean, grapeberry, zucchini and chicory [5], [16]–[17], [19], [39] Expression of homologues of *UBQ* was also found to be stable when evaluated in chrysanthemum and banana under various stresses [11], [38].

Housekeeping genes like *GAPDH* and *ELFA* were also not found to be expressed stably across the diverse experimental sets of *B. juncea*, tested in the current study. The expression of *GAPDH* and *ELFA* was only found to be stable in total development and vegetative stages, respectively (Figure 3; Table 3). Earlier studies have shown that *GAPDH* has constant expression in total developmental stages of chickpea and sugarcane, while it was observed as the least stable transcript in wheat developmental series [10], [21], [40]. Similarly, *ELFA* was also reported as one of the best reference control gene across vegetative stages of rice, perennial ryegrass, chickpea, *Linum usitatissimum, Brachypodium distachyon,* Arabidopsis and chicory [5], [10], [16], [23], [41]–[43]. However, there are evidences that the expression profile of *ELFA* was not as consistent as that of other reference genes in soybean, *Salvia miltiorrhiza* and tomato [17], [18], [44]. The poor performance of *ACT2* across total development, reproductive stages, treatments and cultivars of *B. juncea* was surprising since this gene has been used as a reference control in earlier gene expression studies [27]–[28]. However, *ACT2* can be used in combination of other selected genes for normalization in vegetative stages. Recently, several studies have also shown that the use of *ACT* for normalization is not reliable in rice [16], potato [8], Arabidopsis [23] and peach [45].

The varied expression profiles of commonly used housekeeping genes may be because they are reported to be involved in many other cellular processes besides their basic cellular metabolic functions. For example, *GAPDH* not only acts as a component of the glycolytic pathway, but it is also involved in other processes such as cell proliferation [46]. Similarly, ubiquitin which primarily participates in proteolytic degradation, also has non-proteolytic functions [47]. The varied expression of actin may be due to its participation in cytoplasmic streaming, cell division and the distribution of the plasma membrane proteins other than being a major component of eukaryotic cytoplasmic microfilaments [48].

The newly selected reference genes of *B. juncea*, are superior to traditional ones in terms of their expression stability. The microarray expression data of *B. rapa* and *B. napus* also showed better expression stability of new reference genes identified, compared to the traditional ones (File S1 and S2). Further, the findings of this study are also in good accordance with the Arabidopsis microarray expression data of these 12 candidate reference genes available at AtGenExpress Visualization Tool portal [3], which compiles the expression profiles of five datasets of Arabidopsis (developmental, hormones, abiotic stress, light and pathogen; FileS3). Finally, using the gene expression profiling of *BjDREB2* in abiotic stressed samples of *B. juncea*, we found that normalization involving the combination of more than one stable reference genes resulted in improved accuracy (Figure 4).

### Conclusion

In the present study, we evaluated the expression stability of 12 candidate reference genes across large number of *B. juncea* samples in an effort to identify a set of stable reference gene(s) for normalization during gene expression studies. Analysis of expression stability using geNorm and NormFinder revealed that the expression of *TIPS-41* and *CAC* are most stable across variable experimental tissues. In addition, data analysis using geNorm suggested that three housekeeping genes (*ELFA*, *UBQ9* and *GAPDH*) can be used in combination with *CAC* and/or *TIPS-41* to calculate the normalization factor based on multiple reference genes. Although no candidate reference gene was constantly superior to the others, our data suggest that the novel genes performed better that commonly used housekeeping genes of *B. juncea*. We conclude that the results outline in the present study will facilitate sensitive and accurate quantification of gene expression in *B. juncea* which could also be extrapolated to related Brassica crops. Further, in the absence of enriched genome and transcriptome information from *B. juncea* and its diploid progenitor parents, the current study will greatly assist the Brassica research community to select a set of novel reference genes which could potentially be used for large arrays of experimental conditions and more-importantly in a cross-species manner.

## Methods

### Plant Materials

Mustard (*Brassica juncea* L. cv. Varuna) was used for the experiments. A total of 12 tissues including six vegetative stages (cotyledons, seedlings, young leaf (20 days post sowing), mature leaf (40 dps), root and stem) and six reproductive stages (bud, flower, pod 5 dpa (days post anthesis), pod 10 dpa, pod 15 dpa and pod 30 dpa) were collected from the plant growing in field condition.

Five *B. juncea* cultivars were also included in the study: Early Heera2 (EH2), Pusa Bold (PB), Kranti (KR), Donskaja (DK) and Zem 84500 (ZM). Five vegetative stages (cotyledons, seedlings, leaf, stem and roots) were harvested from these cultivars.

Stress treatments: For stress treatments, 7-day old seedlings were used. Seeds were grown in half strength Murashige-Skoog (MS) media till five days. Elicitors were added after adapting 6-day old seedlings in sucrose free liquid medium for 24 h in dark. Thereafter, seedlings were transferred to beakers containing MS along with sodium chloride (NaCl, 300 mM), abscisic acid (ABA, 100 µM), methyl jasmonate (MeJa, 200 µM), salicylic acid (SA, 200 µM), glucose (3%), indole-3-acetic acid (IAA, 100 µM) and 1-aminocyclopropane-1-carboxylate deaminase (ACC,100 µM), and incubated for 6 h.

For drought treatment seedlings were air dried for 6 h duration; for cold and heat shock treatments the seedlings were kept at 4±1°C and 42±1°C respectively, for 6 h. For wound treatment seedlings were wounded with blunt forceps and collected after 10 min. The mock treated seedling for same interval served as control.

Totally, the experimental samples comprised of 12 developmental stages, 12 exposed to various stress treatments and 25 samples of vegetative stages involving five different cultivars, thereby consisting a total of 49 different tissues.

### RNA Isolation, Quality Control and cDNA Synthesis

RNA was extracted from all the plant tissues using the Spectrum Plant Total RNA Kit (Sigma Life Sciences, USA) according to manufacturer’s instructions. The quantity and quality of RNA sample was checked using Nano spectrophotometer (ND-1000 Thermo scientific); and all RNA samples were adjusted to the same concentration. RNA quality was further assessed using the Agilent-2100 Bioanalyzer and RNA 6000 Nano chips (Agilent Technologies, Singapore). RNA samples with 260/280 ratio from 1.9 to 2.1, 260/230 ratio from 2.0–2.5 and RIN (RNA integrity number) more than 7, were used for further analysis (File S9). The integrity of RNA samples were also checked by agarose gel electrophoresis. For each method the measurement was done in duplicates.

First strand cDNA was synthesized by reverse transcribing 2 µg of total RNA with high-capacity cDNA Reverse Transcription kit (Applied Biosystems, USA) in a 20 µl reaction using mixture of random primers and oligo-dT’s in 1∶1 ratio according to manufacturer’s instructions. cDNA was diluted 50 times for the use of real-time qRT-PCR reaction. All cDNA were stored at −20°C until PCR.

### Selection of Reference Genes and Primer Designing

The five traditional housekeeping genes often used as references control in plants were selected: *ACT* (Actin2, At3G46520), *UBC9* (Ubiquitin9, At4G27960), *ELFA* (Elongation factor 1B, At1G09640), *TUA* (α-Tubulin, At5G19770), *GAPDH* (Glyceraldehyde-3-phospho dehydrogense, At3G04120).

In addition, seven new reference control genes were selected using the available *B. rapa* www.brassica.info/resource/trancriptomics.php [30] and *B. napus* www.rapeseed.plantsignal.cn [31], [32] microarray experiments, covering various abiotic stresses and developmental stages. The ratio of expression levels between control and treatment experiments for each gene that was within the limit of two-fold were selected as putative candidate reference genes. Based on the stable expression profiles during abiotic stress and development stages in *B. rapa* microarray transcriptome data, sequences of four potential new reference genes: *ACP* (Acyl carrier proteins, Arabidopsis ortholog At1G54630), *SNF* (Sucrose non fermenting-1 protein kinase, At3G50500), *TMD* (Leucine rich trans membrane domain proteins, At5G22600), *TIPS-41* (Tonoplastic intrinsic proteins, At4G34270) were selected (File S1). The three remaining genes, *CAC* (Clathrin adaptor complex, At5G46630), *TSB* (Tryptophan synthase-β, At4G27070) and *ZNF* (Zinc finger protein, At1G01930) were selected on the basis of non-significant change in their expression profiles during seed development in *B. napus* transcriptome analysis (File S2).

The full length complementary DNA (CDS) of the Arabidopsis genes were used to query homologous *Brassica* sequences. Expressed sequence tags (ESTs) and Genome survey sequences (GSS) were obtained from the publically available platform at NCBI, Brassica genome gateway http://brassica.bbrc.ac.uk [34] and recently available *B. rapa* genome sequence portal http://www.brassica-rapa.org [49]. The primer for *DREB2* (dehydration responsive binding element) were designed using CDS of Arabidopsis *DREB2* gene and its *B. rapa* gene orthologous (Bra005852 and Bra009112; available on Brassica database http://brassicadb.org [50]. MegAlign module of DNASTAR was used to align the sequenced Brassica EST’s/GSS’s with the known Arabidopsis CDS. The qRT-PCR primer was designed from the consensus sequences preferably spanning the intron(s) (File S4). The details of primer sequences are given in Table 1.

For all genes, primer pairs were designed using the online available Sigma DNA Calculator with the following parameters: optimal length 20–25 nucleotides, melting temperature 60–65°C, GC content <50%, product size range 80–200 base pairs, minimum or no self complementarities at 3′ end, absence for the hairpin structures and self-dimers. In order to confirm the sequences of the amplicons, PCR was performed on cDNA for all designed primer pairs. The products were analyzed on 2% agarose gel and sequenced (File S7). A series of 10 fold of three dilutions of cDNA (10–1,000 fold dilution), were made to determine the gene specific PCR amplification efficiency for each primer pair in qRT-PCR experiments. Based on the Ct values for all dilution points in a series, a standard curve was generated using linear regression and the slope. The qbase http://medgen.ugent.be/~jvdesomp/genorm [51] calculated the gene specific PCR amplification efficiency of the primer using the following equation: Efficiency %  = 10^(−1/slope)^×100%.

Mean of expression levels for all the 12 genes studied in 49 tissue samples were calculated. Standard deviation was calculated using Microsoft Excel and Co-variance was calculated as Standard Deviation/Mean ×100.

### Real-time qRT-PCR Assays

Real-Time PCR was performed in an optical 96-well plate with an 7900 HT real time PCR machine (Applied Biosystems) and universal cycling conditions (95°C for 10 min, 40 cycles of 15 s at 95°C and 60°C for 60 s) in final volume of 20 µl. Reactions contained SYBR Green Master Mix (Kapa Biosystems), 10 pM of a gene specific forward and reverse primers and 2 µl of the diluted cDNA. A no template control (NTC) was also included in each run for each gene; in this study 2 µl RNase free water was used. Each experiment was conducted in three technical replicates with at least two biological replicates for each tissue. To check for the specificity of PCR amplification dissociation curve was generated. The Ct values were automatically determined for each reaction using SDS version 2.3 and RQ manager version 1.2 (Applied Biosciences) software with default parameters.

### Statistical Analysis

Following PCR data collection, two publically available software tools, called geNorm v3.2 [5] and NormFinder v0.953 [3] were used to rank the expression stability of reference genes across all the five experimental sets. The procedures outlined in the user’s manuals, http://medgen.ugent.be/~jvdesomp/genorm [51] and http://www.mdl.dk/publicationsnormfinder.htm [52], were followed to calculate instability values of gene expression.

Briefly, the geNorm program is based on pairwise comparisons and stepwise exclusion of candidate genes according to their expression stability measures (M) values. In general, lower the M value, higher the gene expression stability. geNORM recommends M<1.5 to identify sets of reference genes with stable expression. Further, the pairwise variation (Vn/Vn+1) between sequential normalization factors, NFn and NFn+1 was used to determine the number of genes required for reliable normalization. A threshold value of 0.15 was reported by Vandesompele et al. [6] for normalization of expression stability. It has been suggested that below this threshold values there is no need of an additional internal control gene.

The NormFinder reference tool was also applied to rank the candidate reference genes expression stability for all the samples with no subgroup determination. NormFinder used ANOVA based model to estimate intra- and inter-group variation, and combines these estimates to provide a direct measure of the variations in expression for each gene [4]. It ranks the genes according to their stability under given set of experimental conditions. Genes with lower average expression stability values are more stable.

## Supporting Information

File S1 Gene expression profile of the genes considered in present study, as observed in *B. rapa* microarray database (http://www.brassica-rapa.org).
(XLS)

File S2 Expression profile of the selected genes as observed in previous study by Niu et al., 2009 [31] on *B. napus* seed development and fatty acid metabolism (www.rapeseed.plantsignal.cn).
(XLS)

File S3 Arabidopsis microarray expression data of development stages showing the expression profile of 12 candidate reference genes considered in this study (available at jsp.weigelworld.org).
(PPT)

File S4 An example of DNA sequence alignment of Arabidopsis CDS and its homologous Brassica EST and GSS. The nucleotide bases highlighted in grey are the conserved sequences for designing primers for *TIPS*-41.
(PPT)

File S5 The confirmation of expected amplicon size of the primer pairs.
(PPT)

File S6 Melt curves of the candidate reference genes under different experimental conditions.
(PPT)

File S7 Sequencing data of the PCR amplicons.
(DOC)

File S8 Amplification efficiencies of the primers designed in the current study.
(PPT)

File S9 The integrity values of RNA samples determined using Bioanalyzer.
(PPT)
