# Supplementary material for: Evaluation of Candidate Reference Genes for Gene Expression Normalization in Brassica juncea Using Real Time Quantitative RT-PCR
Source: PLoS One. 2012 May 11;7(5):e36918. doi: 10.1371/journal.pone.0036918 (PMC3350508; doi:10.1371/journal.pone.0036918)
Supplement: File S3 — Arabidopsis microarray expression data of development stages showing the expression profile of 12 candidate reference genes considered in this study (available at jsp.weigelworld.org). (PPT) [file pone.0036918.s003.ppt]

## Slide 1
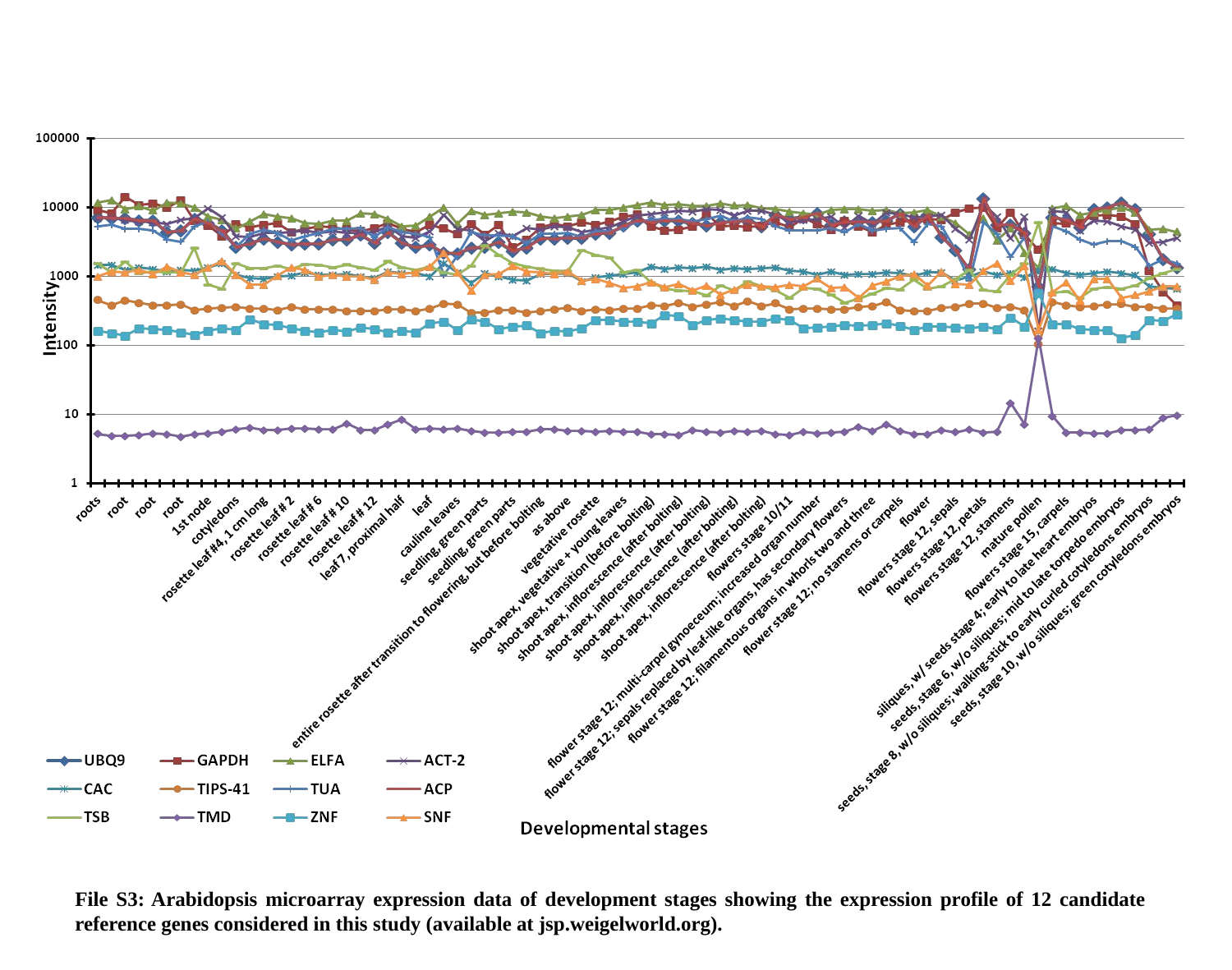

File S3: Arabidopsis microarray expression data of development stages showing the expression profile of 12 candidate reference genes considered in this study (available at jsp.weigelworld.org).
