# Supplementary material for: Evaluation of Candidate Reference Genes for Gene Expression Normalization in Brassica juncea Using Real Time Quantitative RT-PCR
Source: PLoS One. 2012 May 11;7(5):e36918. doi: 10.1371/journal.pone.0036918 (PMC3350508; doi:10.1371/journal.pone.0036918)
Supplement: File S4 — An example of DNA sequence alignment of Arabidopsis CDS and its homologous Brassica EST and GSS. The nucleotide bases highlighted in grey are the conserved sequences for designing primers for TIPS-41. (PPT) [file pone.0036918.s004.ppt]

## Slide 1
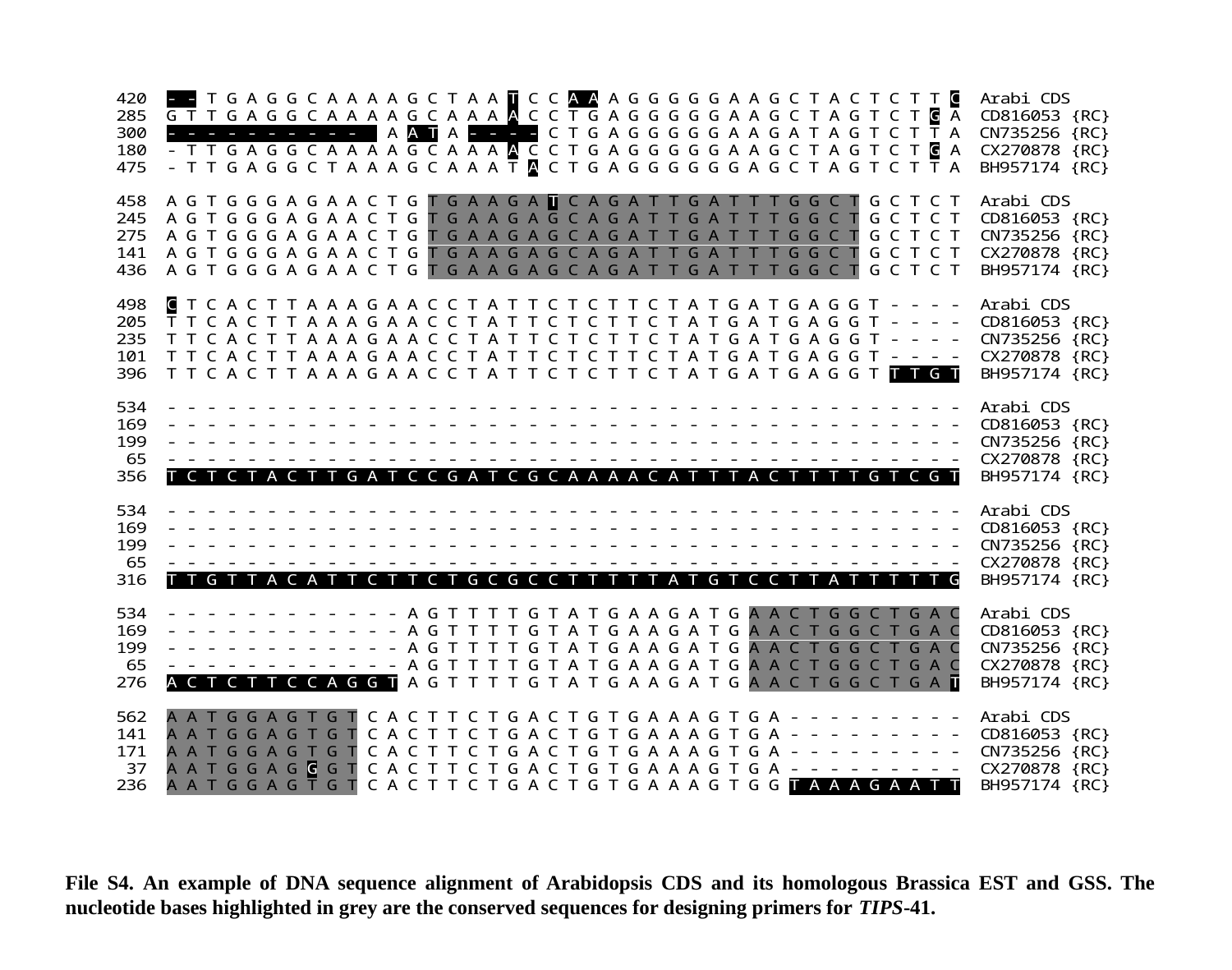

File S4. An example of DNA sequence alignment of Arabidopsis CDS and its homologous Brassica EST and GSS. The nucleotide bases highlighted in grey are the conserved sequences for designing primers for TIPS-41.
