# Supplementary material for: Evaluation of Candidate Reference Genes for Gene Expression Normalization in Brassica juncea Using Real Time Quantitative RT-PCR
Source: PLoS One. 2012 May 11;7(5):e36918. doi: 10.1371/journal.pone.0036918 (PMC3350508; doi:10.1371/journal.pone.0036918)
Supplement: File S5 — The confirmation of expected amplicon size of the primer pairs. (PPT) [file pone.0036918.s005.ppt]

## Slide 1
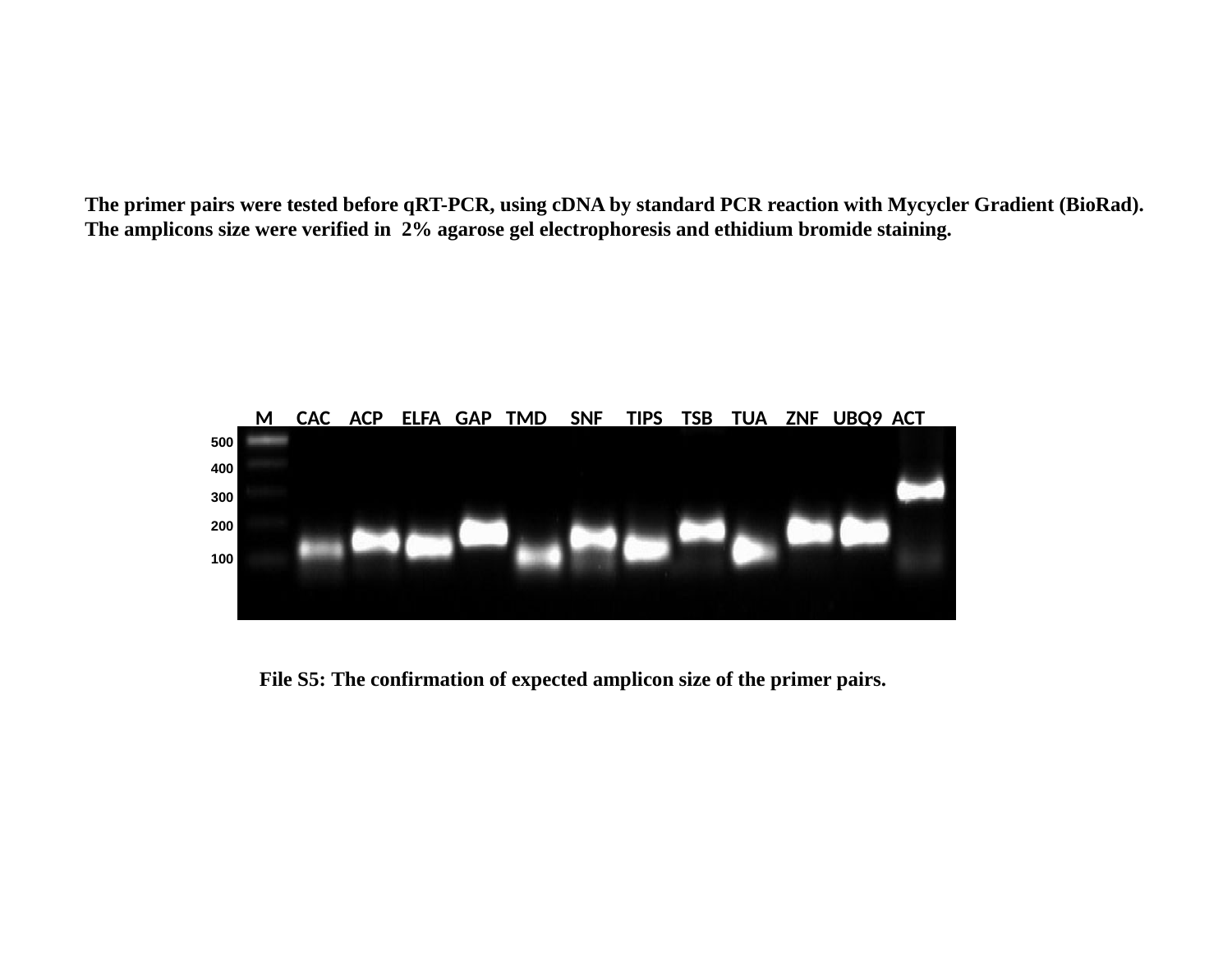

The primer pairs were tested before qRT-PCR, using cDNA by standard PCR reaction with Mycycler Gradient (BioRad). The amplicons size were verified in 2% agarose gel electrophoresis and ethidium bromide staining.
M CAC ACP ELFA GAP TMD SNF TIPS TSB TUA ZNF UBQ9 ACT
500
400
300
200
100
File S5: The confirmation of expected amplicon size of the primer pairs.
