# Supplementary material for: Evaluation of Candidate Reference Genes for Gene Expression Normalization in Brassica juncea Using Real Time Quantitative RT-PCR
Source: PLoS One. 2012 May 11;7(5):e36918. doi: 10.1371/journal.pone.0036918 (PMC3350508; doi:10.1371/journal.pone.0036918)
Supplement: File S6 — Melt curves of the candidate reference genes under different experimental conditions. (PPT) [file pone.0036918.s006.ppt]

## Slide 1
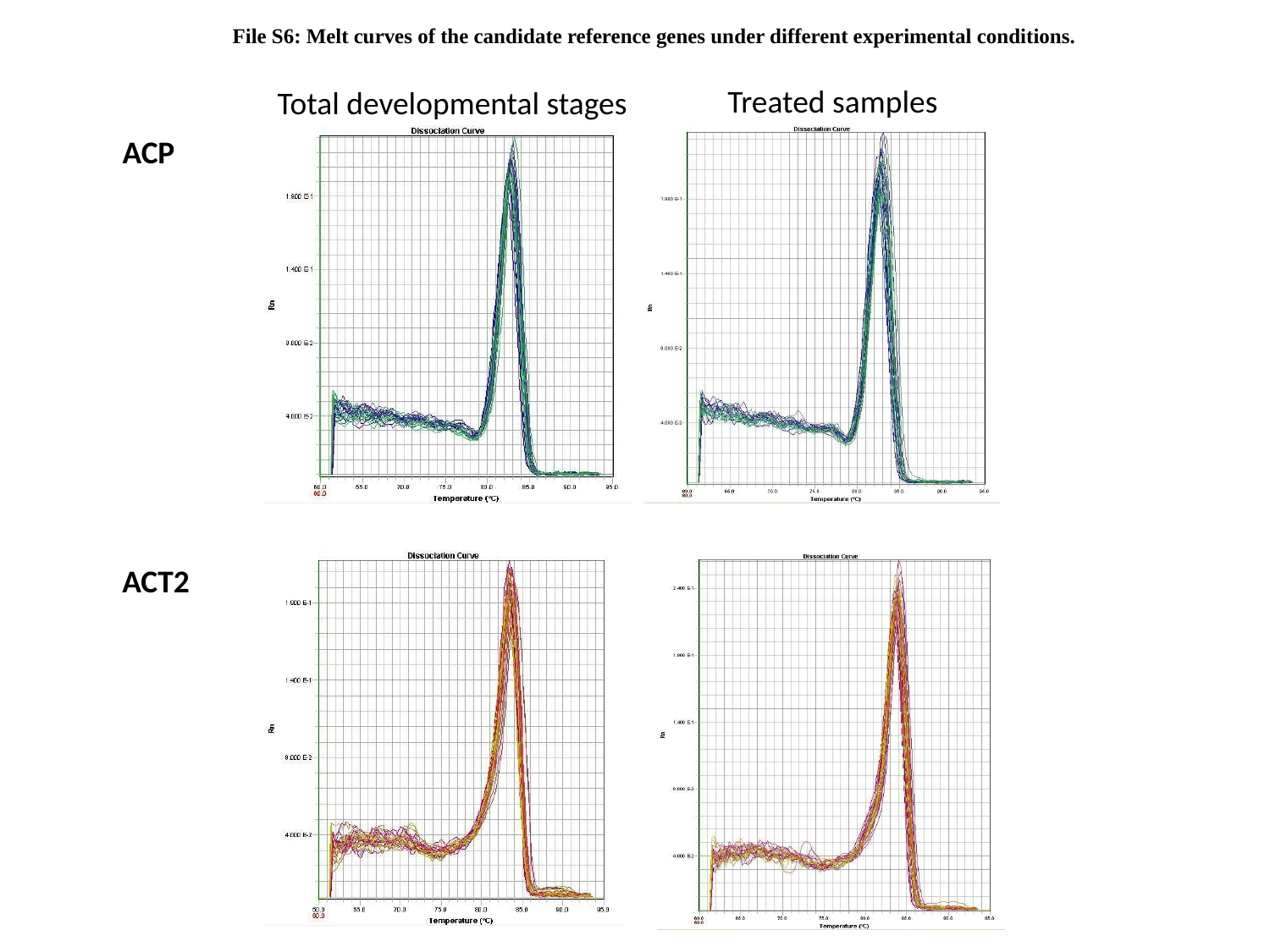

File S6: Melt curves of the candidate reference genes under different experimental conditions.
Treated samples
Total developmental stages
ACP
ACT2

## Slide 2
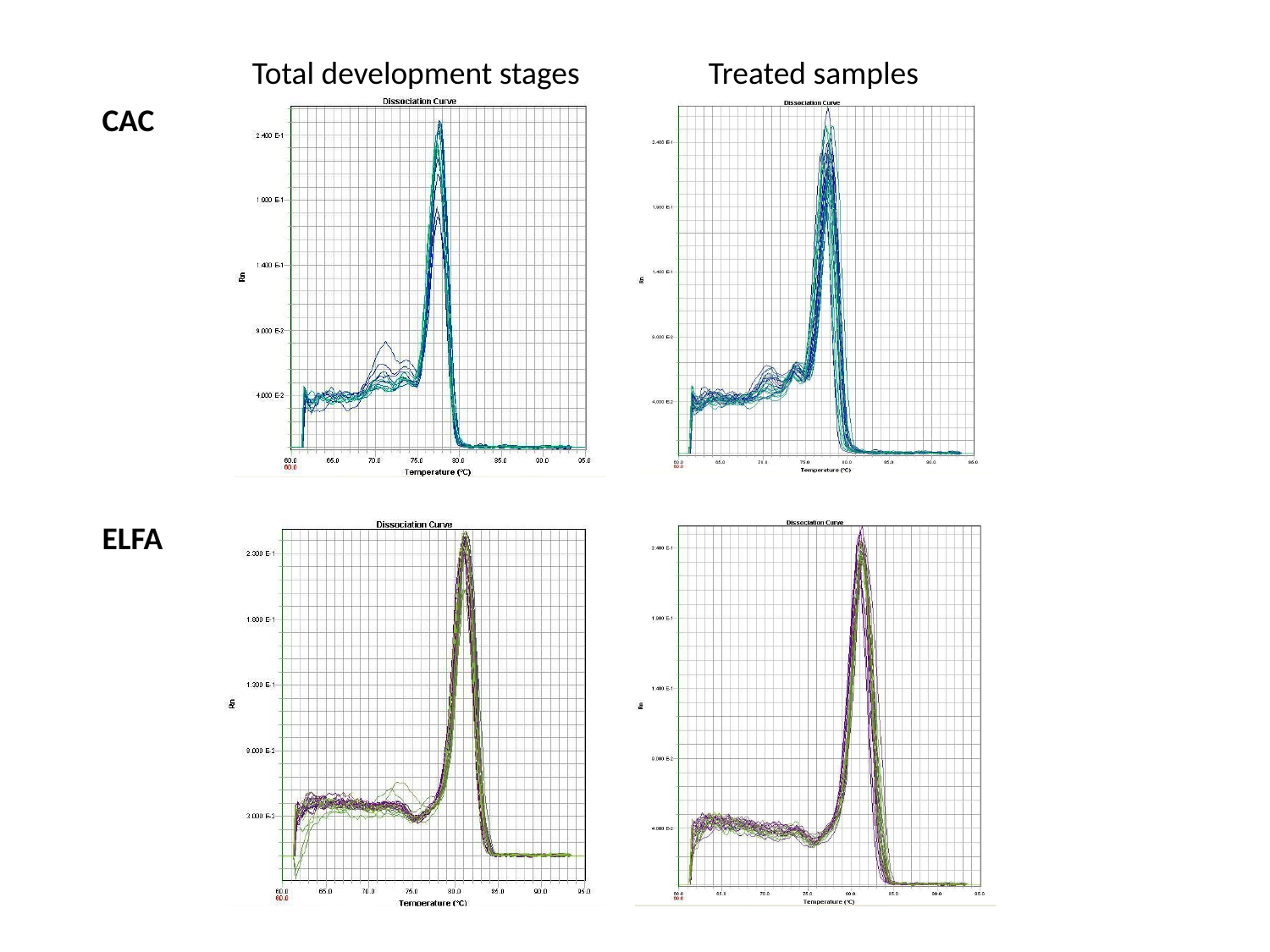

Total development stages
Treated samples
CAC
ELFA

## Slide 3
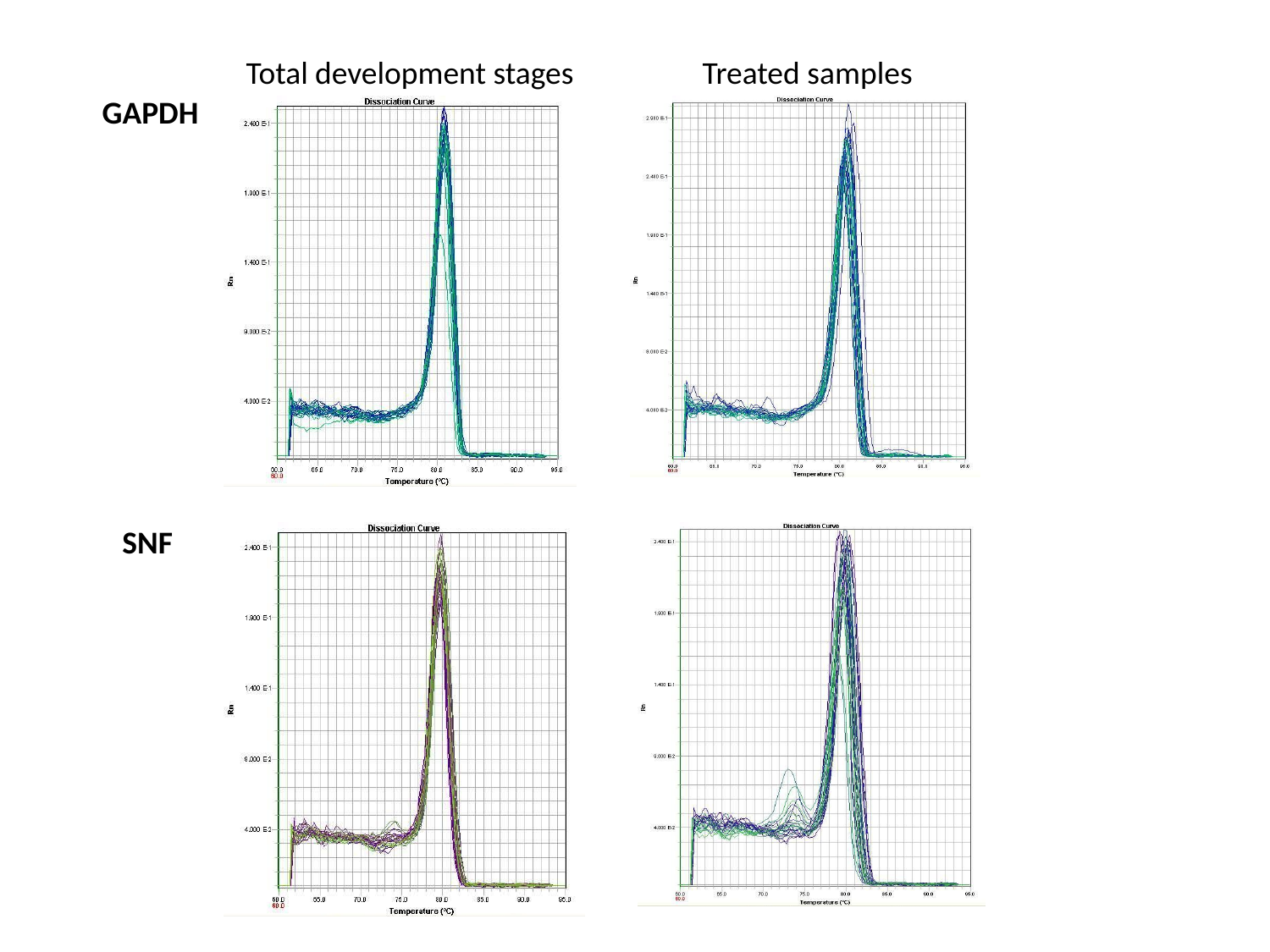

Total development stages
Treated samples
GAPDH
SNF

## Slide 4
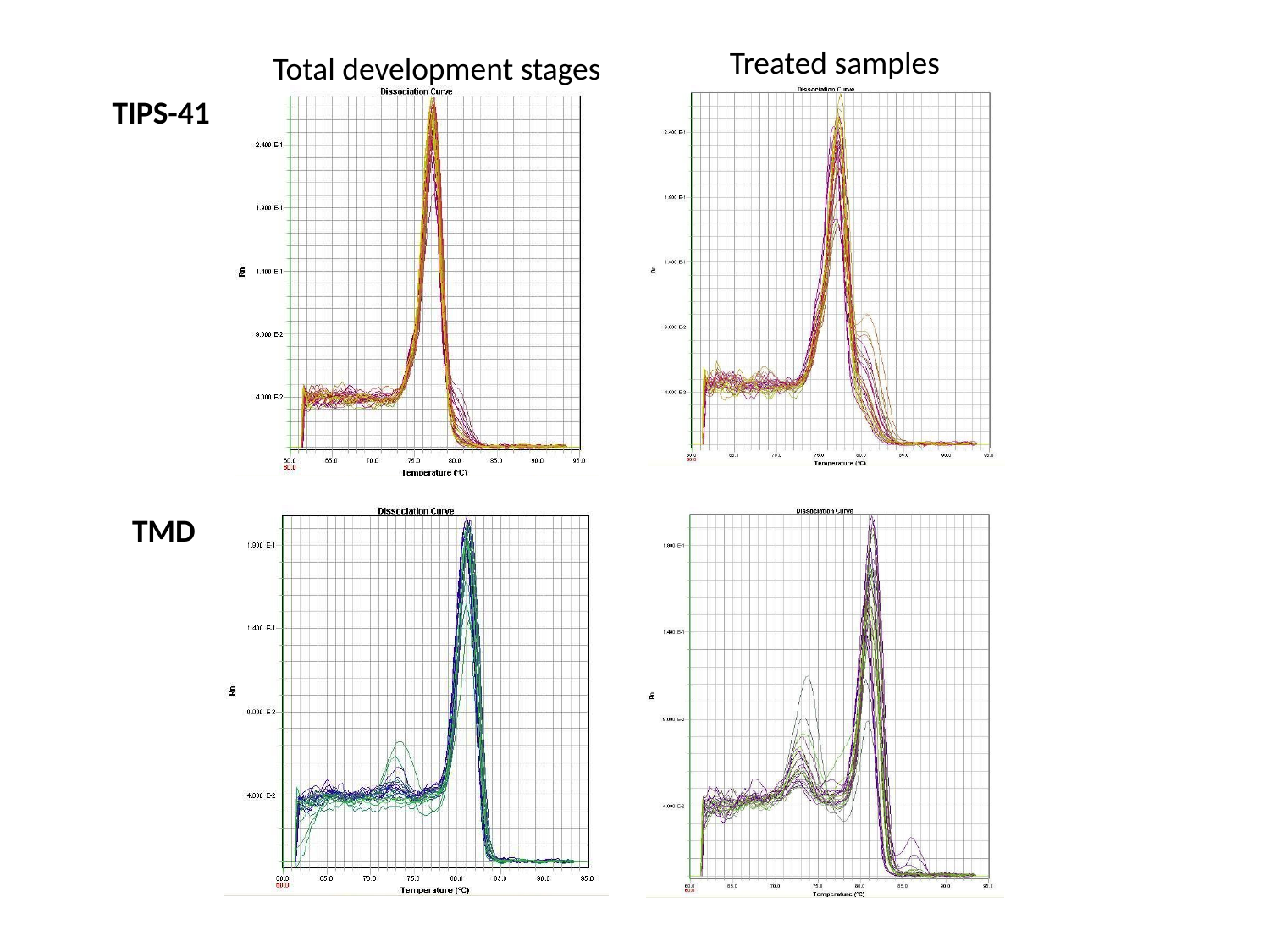

Treated samples
Total development stages
TIPS-41
TMD

## Slide 5
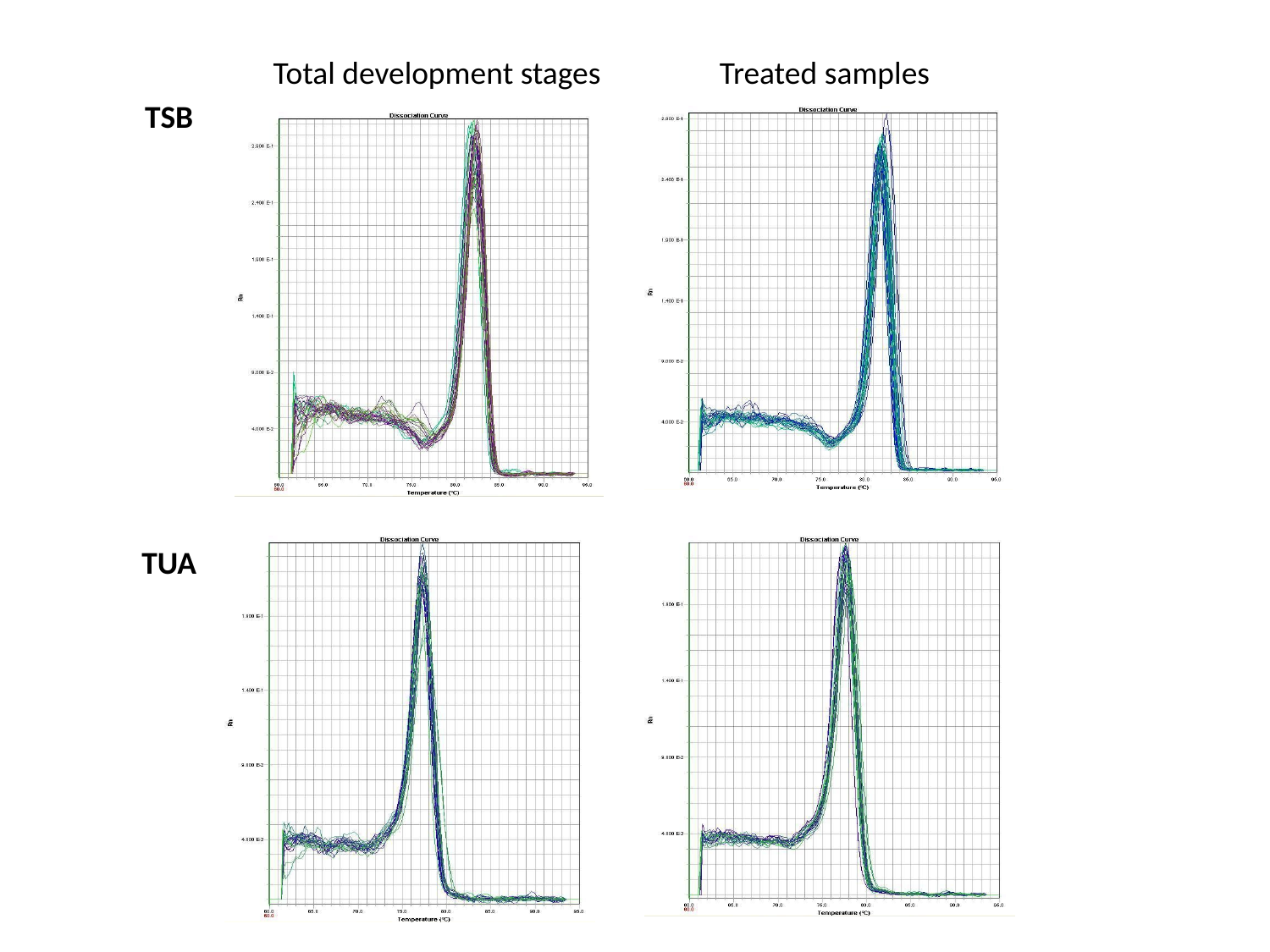

Total development stages
Treated samples
TSB
TUA

## Slide 6
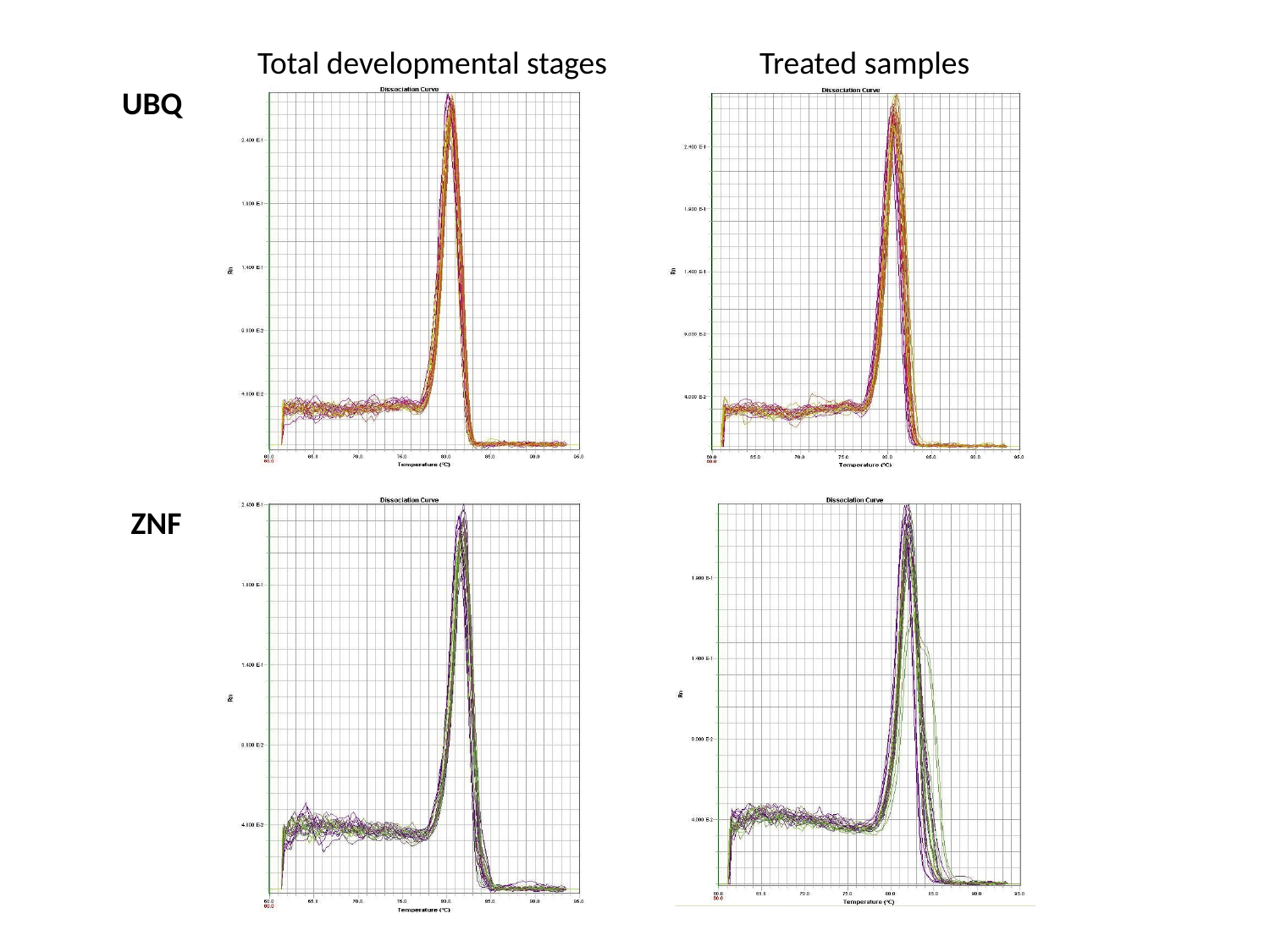

Total developmental stages
Treated samples
UBQ
ZNF
