# Supplementary material for: Evaluation of Candidate Reference Genes for Gene Expression Normalization in Brassica juncea Using Real Time Quantitative RT-PCR
Source: PLoS One. 2012 May 11;7(5):e36918. doi: 10.1371/journal.pone.0036918 (PMC3350508; doi:10.1371/journal.pone.0036918)
Supplement: File S7 — Sequencing data of the PCR amplicons. (DOC) [file pone.0036918.s007.doc]

| **Gene** | **Sequence** |
| --- | --- |
| ACP | TGTTTTAGCCAGGGAAGCAGAAGCAATCTTTCTTTTACGCTTCGCCCTCTTCCTACCCGCCTGAGCGTTTCTTGCGCTGCAAAACCTGAGACAGTGGACAAAGTGTGT |
| ACT | TAACCCAAAGGCCAACAGAGAGAAGATGACCCAGATCATGTTCGaAACCTTCAACTCTCCAGgCTATGTATGTCGCYATCCAAGCTGTTCTCTCCCTGTACGCCAGTGGTCGTACTACCGGTATTGTGCTGGATTCTGGTGATGGTGTGTCTCACACTGTGCCAATCTACGAGGGTTTCTCTCTTCCACACGCCATCCTCCGTCTCGACCTTGCTGGTCGTGACCTTACTGATT |
| CAC | CAATCGATTGCTTGGTTTGGAAGATAAGAAAATTTCCAGGACAAACAGAGTCCACGCTAAGTGCAGAGATTGAGTTGATCTCAACAATGGGAGAGAAGAAATCTTGGACAAGGCC |
| ELFA | GGTTCGTCCCAAGAATGGGCTTTATGCCTTACAGTGTTCCGGGTGAGGAAGCTGCCATCTCTGCTTTGAAAAGAGGACTTGATGCACTGAACACACACCTCGCCTCCAACACTTACCTCGTTGGA |
| GAPDH | TGTCTCAGTTGTTGACCTCACGGTTAGACTCGAGAGAAAGCTGCAACCTACGACGAGATCAAGAAGGCTATCAAGGAGGAATCTGAAGGCAAGCTAAAGGGAATCCTTTGGTTACACCGAGGATGATGTTGTCTCA |
| SNF | CAAAGTCAACTGTTGGTACTCCGCATACATCGCAGGAGAGATCTTCTTGACAGGAATATGATGGCAAGATAGATGGCAGATGTATGGTCCTGTGGTGTAACCTTATACGTAATGTTGGTTGATGATGCATATCCA |
| TIPS | GTGAAGAGCAGATTGATTTGGCTGCTCTTTCACTTAAAGAACCTATTCTCTTCTATGATGAGGTAGTTTTGTATGAAGATGAACTGGCTGATAATGGAGGGTCACTTCTGACTGTGAAAGTGA |
| TMD | CGTACTCAATTCTATCTCCGCCTCTTTTCCTCATCTCCCCGCCAAATTCGACGGCTCCGTCCCCCAGAAACGGAATATGCGGAGCTCTGTTCGTCGCA |
| TSB | TGTCGCTGACTTGAAGTACGGTCCCCGAAACTCTAATGCACGCCTCTCCGAACTCGAAACTGCTTTCTACTCGCTTGCCACCGACGATGATTTCCAGAGGGAGTTAGCTGGGATCTTGAGGATCTTGAAAGACTACGTGGCTCGTGAAAGGATCTTGAAA |
| UBQ | GAAGACATGTTCCATTGGCAAGCCACGATAATGGGTCCATCCGACAGCCCTTACTCTGGTGGCGTTTTTCCTTGTTACCATTCATTTCCCTCCTGATTATCCTTTTAAACCTCCTAAGGTGGCTTTTAGGACTAAGGTGTTCCACC |
| ZNF | CAGGCGGTTTATGGCGTCAAACCTTGAAAAATGGAATTGGCATGATGCTAAAGTACCCAGCCCGCTCACCAAGGAGATGGAGGAATCTCAAGCTGCTAAGCAGGCAGAGAAAGACGCCAAGAAGAAAGCAAGAGCAAA |

**File S7: Sequencing data of the PCR amplicons.**
