# Supplementary material for: Evaluation of Candidate Reference Genes for Gene Expression Normalization in Brassica juncea Using Real Time Quantitative RT-PCR
Source: PLoS One. 2012 May 11;7(5):e36918. doi: 10.1371/journal.pone.0036918 (PMC3350508; doi:10.1371/journal.pone.0036918)
Supplement: File S8 — Amplification efficiencies of the primers designed in the current study. (PPT) [file pone.0036918.s008.ppt]

## Slide 1
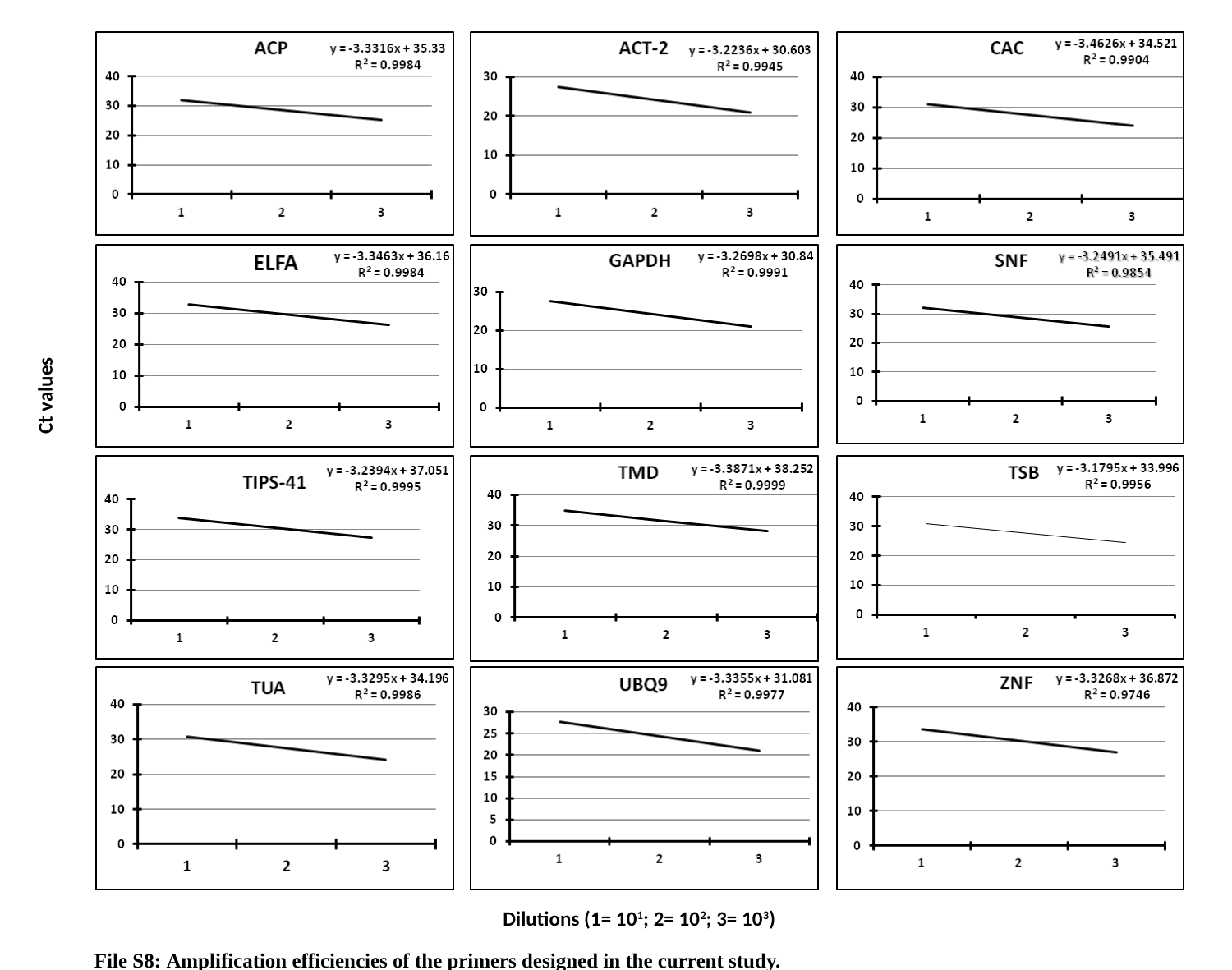

Ct values
Dilutions (1= 101; 2= 102; 3= 103)
File S8: Amplification efficiencies of the primers designed in the current study.
