# Supplementary figures and images for: Evaluation of Candidate Reference Genes for Gene Expression Normalization in Brassica juncea Using Real Time Quantitative RT-PCR
Source: PLoS One. 2012 May 11;7(5):e36918. doi: 10.1371/journal.pone.0036918 (PMC3350508; doi:10.1371/journal.pone.0036918)

## Slide 1
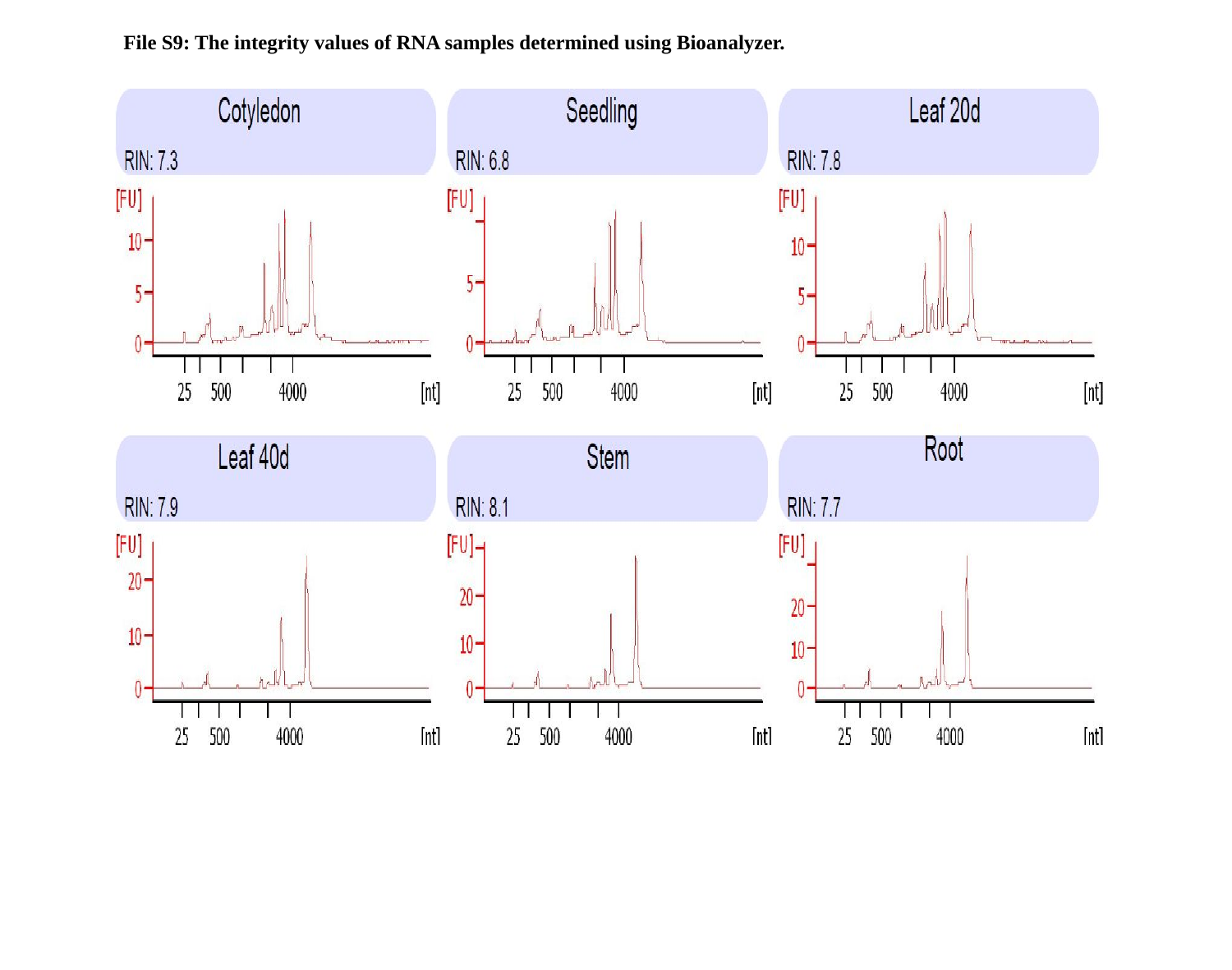

File S9: The integrity values of RNA samples determined using Bioanalyzer.

## Slide 2
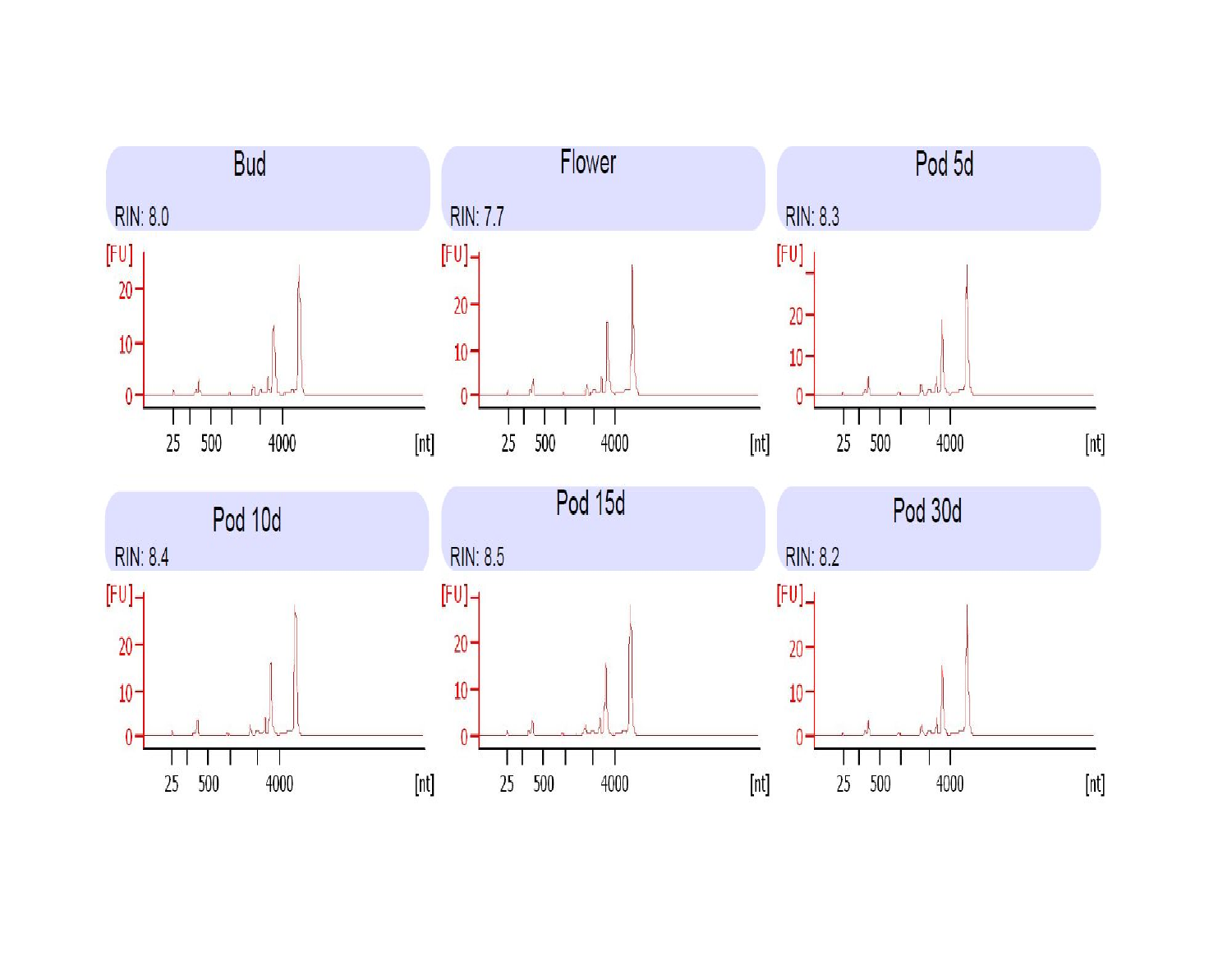

Supplement: File S9 — The integrity values of RNA samples determined using Bioanalyzer. (PPT) [file pone.0036918.s009.ppt]
